# Climate Change: A National Survey on Viewpoints of Italian Epilepsy‐Care Professionals

**DOI:** 10.1111/ene.70684

**Published:** 2026-08-21

**Authors:** Emanuele Bartolini, Simona Balestrini, Giulia Battaglia, Pia Bernardo, Giovanni Falcicchio, Francesco Fortunato, Manuela Lo Bianco, Silvia Pradella, Teresa Ravizza, Alessandra Rossi, Leila Zummo, Annamaria Vezzani, Emilio Russo, the Italian League against Epilepsy (LICE) Climate Change Study Group

**Affiliations:** ^1^ Department of Developmental Neurosciences IRCCS Stella Maris Foundation Pisa Italy; ^2^ Neuroscience and Human Genetics Department Meyer Children's Hospital IRCCS, University of Florence Florence Italy; ^3^ Department of Neuroscience, Pharmacology, and Child Health University of Florence Florence Italy; ^4^ Research Department of Epilepsy UCL Queen Square Institute of Neurology London UK; ^5^ Epilepsy Unit Fondazione IRCCS Istituto Neurologico Carlo Besta Milan Italy; ^6^ Department of Neurosciences, Pediatric Neuropsichiatry Unit Santobono‐Pausilipon Children's Hospital, AORN Naples Italy; ^7^ Department of Translational Biomedicine and Neurosciences (DiBraiN) University of Bari Bari Italy; ^8^ Department of Medical and Surgical Sciences, Institute of Neurology Magna Graecia University Catanzaro Italy; ^9^ Unit of Pediatric Clinic, Department of Clinical and Experimental Medicine, A.O.U. Policlinico G. Rodolico‐San Marco University of Catania Catania Italy; ^10^ Neurology Unit USL Tuscany Center Prato Italy; ^11^ Department of Acute Brain and Cardiovascular Injury Istituto di Ricerche Farmacologiche Mario Negri IRCCS Milan Italy; ^12^ Department of Pediatrics Ospedale Santo Spirito, Azienda Sanitaria Pescara Pescara Italy; ^13^ Neurology and Stroke Unit P.O. ARNAS‐Civico Palermo Italy; ^14^ CRUISE Research Center, Department of Health Sciences University Magna Graecia Catanzaro Italy

## Abstract

**Objective:**

To assess awareness, attitudes, and preparedness among Italian epilepsy‐care professionals regarding the impact of climate change on epilepsy management.

**Methods:**

We conducted a national, anonymous Italian‐language online survey distributed through professional networks and scientific societies (LICE, SNO). The questionnaire included Likert‐scale items, closed‐ended responses were summarized descriptively.

**Results:**

We collected 167 responses from a heterogeneous sample by professional role and geographical area. All respondents acknowledged that global warming is occurring; 100% rated its impact on future generations as moderate‐to‐severe, and 84.3% reported moderate‐to‐severe concern for their patients. Support was high for continuing professional education on climate and health (99.4%), patient educational materials (88.6%), and workplace sustainability guidelines (97%). Individual mitigation actions were common (e.g., 93.3% reported recycling more frequently), whereas institutional initiatives were less consistently reported (e.g., 46.1% reported telemedicine use and 42.6% reported no transportation‐related initiatives).

**Conclusions:**

Italian epilepsy‐care professionals demonstrate high awareness and concern regarding climate change and its impact on patients. While support for education and sustainability initiatives is strong, translation into institutional and research practices remains limited, highlighting the need for targeted policies and infrastructure.

## 1 Introduction

Climate change is a major and escalating threat to environmental and human health. The human brain is highly sensitive to thermal and metabolic stress, making individuals with neurological disorders—particularly those with epilepsy—especially vulnerable. Some individuals with genetic epilepsies may have ion‐channel mutations that are temperature‐sensitive (e.g., in febrile seizures, heat‐sensitive epilepsies such as Dravet syndrome)—thus, ambient conditions may tip the balance [1]. However, climate‐related stressors may also affect people with non‐genetic epilepsies through physiological, behavioral, and health‐system pathways. Epilepsy is a common chronic neurological condition with substantial morbidity and impact on quality of life; therefore, even modest climate‐related changes in seizure control could have important clinical and societal consequences. Available evidence, although still limited, supports an association between meteorological conditions and seizure occurrence. High ambient temperature or heat stress in animal models can elevate core and brain temperatures and enhance neuronal excitability, thereby lowering seizure thresholds [1]. However, systematic studies in humans are still lacking. A small‐scale study of nine people undergoing intracranial EEG found more seizures during heatwave periods (≥ 3 consecutive days > 28°C) compared to non‐heatwave periods [2]. High temperature is not the only risk factor. Low pressure, high humidity—often occurs during storms or “bad weather” fronts—significantly increases the risk of seizures [3]. Systemic imbalances may also be implicated. During hot/humid weather, dehydration or sweating can lead to hyponatremia, hypoglycaemia, or other electrolyte shifts—all of which can trigger seizures in susceptible individuals [4]. Extreme weather may promote infections or vector‐borne diseases, which can include fever as a trigger for seizures [5]. Changes in the gut microbiome may represent a biological mechanism through which seizure burden worsens. Evidence connects gut dysbiosis to neuroinflammation and epilepsy, while separate research demonstrates that climatic and meteorological factors can perturb both the gut microbiome and immune function [6, 7]. Although direct human data linking climate‐driven dysbiosis to seizure exacerbation are currently lacking, this pathway remains a biologically plausible hypothesis warranting further investigation [7]. Indeed, seizure precipitants such as sleep deprivation, stress, and fatigue may also be favored by climate change. Sleep quality may be lowered by the elevation of nocturnal temperatures. Stress and fatigue may result from coping with uncomfortable environmental conditions. Psychosocial stressors related to climate change—including anxiety, social isolation, and climate‐related distress—may further worsen seizure control and quality of life. Disruptions in medical care (e.g., power outages, medication storage, and stability during heat/humidity) may indirectly raise seizure risk [1, 5]. Some work suggests that lower temperatures (or sudden temperature drops) may be harmful. One study found that every 1°C drop in temperature increased the relative risk of an emergency hospital visit for epilepsy by ~1.6% [8]. Both heat and cold extremes (and rapid changes) may act as stressors and lower seizure thresholds.

These observations underscore the importance of considering environmental factors as integral components in the clinical management of epilepsy. Despite increasing recognition of this issue, little is known about the level of awareness, perceived relevance, and preparedness of healthcare professionals regarding the effects of climate change on epilepsy. Understanding clinicians' knowledge, attitudes, and concerns is essential to identify educational needs and inform the development of adaptive clinical and policy strategies. The present study reports findings from a national survey conducted among healthcare professionals in Italy to assess their awareness and perceptions of the impact of climate change on epilepsy management. The results aim to inform future initiatives in research, clinical practice, and health policy, contributing to the development of evidence‐based recommendations for epilepsy care in the context of a changing climate.

## 2 Methods and Population

We developed an Italian‐language survey to assess awareness, attitudes, and professional activities related to epilepsy and climate change among clinicians and other epilepsy‐related professionals. The survey design was informed by the methodology of Blenkinsop et al. [9], adapting their structure and thematic areas to the Italian professional context. The individual professional characteristics were self‐reported, based on the professional profile of each respondent. We developed a 22‐item questionnaire using REDCap to assess climate change awareness, attitudes, and behaviors among Italian neurology professionals, particularly in epilepsy care contexts. It comprised Likert‐scale items (5‐point agreement/disagreement or utility scales; 11 items) quantitatively measuring beliefs (e.g., human mitigation potential), perceived harms (rated “a lot/moderately/little/not at all/don't know” for self, patients, future generations, and research; 5 items), resource utility (e.g., scientific society statements, CME training, patient materials; 4 items), and support for adaptations (e.g., virtual conferences, fossil fuel divestment; 2 items). These were complemented by multi‐select checklists (6 items) capturing self‐reported behavioral changes (waste/energy/transport/diet/research practices) and institutional green initiatives (e.g., energy conservation, telemedicine), plus demographics (role, age, career stage, gender, region; 3 items). No open‐ended questions were included (available at https://redcap.link/6g6zlc4x). The draft survey was reviewed for clarity and relevance by experts in epilepsy care and environmental health, then distributed electronically through professional networks and national societies. Respondents were invited to participate through a publicly accessible survey link, disseminated by the Italian League Against Epilepsy (LICE) and by the Hospital Neurology Society (SNO). The survey was open between 1/2/2025 and 31/1/2026. No formal validation or psychometric evaluation was performed prior to questionnaire administration, as we aimed to build a hypothesis‐generating instrument. Responses were analyzed using descriptive statistics and reported qualitatively.

To quantify perceptions of climate change according to professional role, career stage, and geographic workplace, we performed a further analysis. Three selected survey items were also operationalized into numerical scores using a linear ordinal scaling approach and named Future Generations (‘How much do you think climate change will harm future generations?’), Personal Impact (‘How much do you think climate change will harm future generations?’), Patient Health (‘How much do you think climate change will harm patients’ health?’). The items were mapped to a 4‐point Likert‐type scale, and negative‐phrased items were reverse‐coded to ensure that higher values consistently represented greater climate awareness or pro‐environmental orientation. A composite Climate Concern Index was subsequently calculated for each respondent by averaging the scores of the three variables. To enhance statistical power for regional comparisons, geographic data were aggregated into three macro‐regions (North, Center, and South/Islands) based on administrative boundaries.

### 2.1 Ethical and Regulatory Approval

We confirm that we have read the Journal's position on ethical publication practices and affirm that this report complies with those guidelines. The work was conducted in Italy as a survey‐based assessment of professional awareness and practices, posing minimal informational or psychological risk. In accordance with national standards for observational, non‐interventional surveys that do not collect identifiable data, the study did not require review or approval by an Ethics Committee. Participation was voluntary and anonymous, and no personal or sensitive information was collected.

### 2.2 Participant Characteristics

We obtained a total of 167 survey responses. The majority of respondents were female (69.0%), followed by male (29.9%), with 0.6% preferring not to answer. Participants represented a range of professional roles, qualifying themselves as adult neurologists (77, 46.7%), epileptologists (51, 30.9%), child neuropsychiatrists (39, 23.6%), basic science researchers (3, 1.8%), pediatricians (9, 5.5%), neuropsychologists (1, 0.6%), neurosurgeons (1, 0.6%), nurses (1, 0.6%), neurophysiology technicians (16, 9.7%), medical residents (10, 6.1%), and other professions (e.g., psychiatrist, social worker, occupational therapist) (6, 3.6%). Participants spanned all age ranges, with 18–24 years (1, 0.6%), 25–34 years (38, 23.0%), 35–44 years (50, 30.3%), 45–54 years (38, 23.0%), 55–64 years (23, 13.9%), and over 65 years (15, 9.1%). In terms of career stage, 50.6% were in advanced stages, 28.7% in mid‐career, and 20.7% in early stages. Geographically, respondents were distributed across Italy, with 37.0% from Northern regions, 43.2% from Central regions, and 22.2% from Southern regions and the islands; one respondent (0.6%) reported working outside Italy.

## 3 Results

**FIGURE 1:**
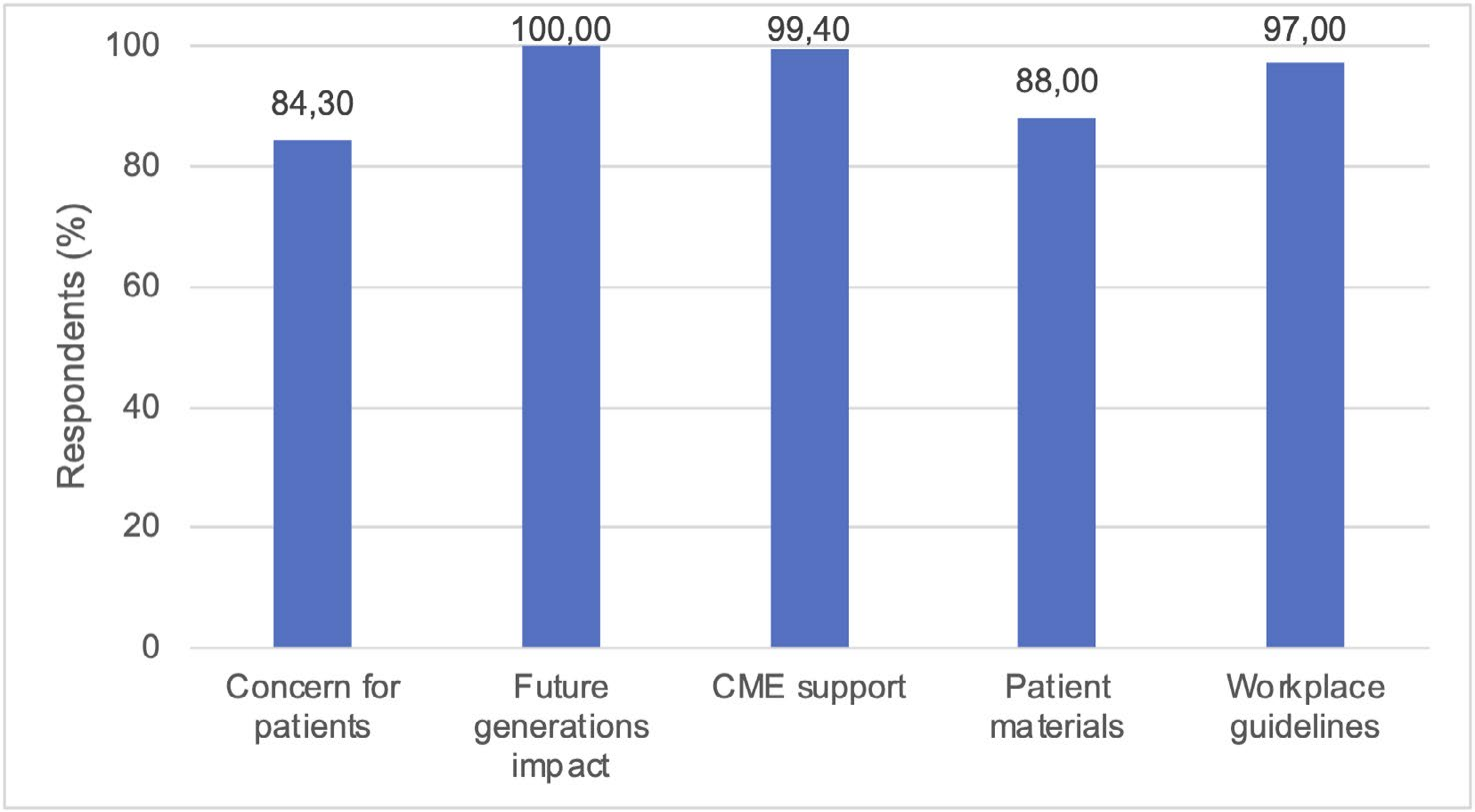
Selected survey findings on climate‐change awareness, perceived impact, and support for professional resources. Bars indicate the percentage of respondents reporting moderate‐to‐severe concern for patients' health, moderate‐to‐severe impact on future generations, support for continuing medical education on climate and health, perceived usefulness of patient educational materials, and support for workplace sustainability guidelines.

A summary of participants' responses is shown in Table 1, and illustrative examples are depicted in Figure 1.

**TABLE 1:** Selected survey findings (*N* = 167).

Item	*n*	%
Belief that global warming is occurring	167	100.0
Moderate‐to‐severe concern for patients	141	84.3
Moderate‐to‐severe impact on future generations	167	100.0
Support continuing professional education on climate/health	166	99.4
Patient educational materials considered useful	148	88.6
Workplace sustainability guidelines considered useful	162	97.0
Recycling more frequently at home	156	93.3
Telemedicine reported as used in the workplace	77	46.1
No transportation‐related initiatives reported in the workplace	71	42.6

*Note:* Percentages are as reported in the survey; counts are rounded to the nearest integer.

### 3.1 Awareness and Perceptions of Climate Change

Out of 167 respondents, none believe that global warming is not happening. A very small proportion (0.6%) think that humans are unable to reduce it. About 28.1% believe that although humans could reduce global warming, people are not willing to change their behavior. The majority (65.3%) think that humans could reduce global warming, but it is uncertain whether this will happen. Only 6.0% of respondents express confidence that humans will both reduce global warming and succeed in doing so. Concerns about climate change are deeply felt among healthcare professionals, with 100% rating its impact on future generations as moderate to severe. On a personal level, 83.9% expressed moderate to severe concern, and similarly, 84.3% were concerned about their patients, with nearly 29% being very much concerned. Half of the respondents believed that their research activities would be negatively affected by climate change. Interestingly, only a minority (13.8%) agreed that individual actions do not make a difference, while a substantial 84.4% reported having personally experienced the effects of climate change.

### 3.2 Professional Resources and Training

Regarding professional resources and training, respondents overwhelmingly considered valuable official statements from scientific societies, with 65.7% considering them very useful. Nearly all (99.4%) supported continuing professional education on climate and health, and 88.6% found patient educational materials useful. Workplace sustainability guidelines had a 97% approval rate. On organizational policies, 60.2% agreed that societies should offer virtual participation in conferences to reduce travel emissions, and 79% supported cutting ties with fossil fuel companies.

### 3.3 Individual Actions in Daily Life

In their daily lives, participants reported numerous personal actions in response to climate change awareness: 93.3% recycle more frequently at home, 64.8% buy products with less packaging, 53.9% compost organic waste, and 61.8% donate or reuse items. Energy‐saving behaviors included purchasing energy‐efficient appliances (73.3%), replacing conventional bulbs with low‐energy alternatives (79.4%), installing solar technologies (37.5%), and using renewable energy suppliers (14.5%). In transportation, 28.4% drove electric or hybrid cars, 31.5% used public transport more often, 48.5% walked or cycled instead of driving, and 46.1% avoided flying when possible. Regarding dietary choices to reduce carbon footprints, about 43.7% reported often or always making low‐carbon choices.

### 3.4 Changes in Research Practices

In research practices, a minority participated in green action groups (5.5%), or climate change training (8.6%), and 12.3% adopted mitigation strategies in everyday research work, whereas 76.7% reported no changes.

### 3.5 Institutional and Workplace Initiatives

**FIGURE 2:**
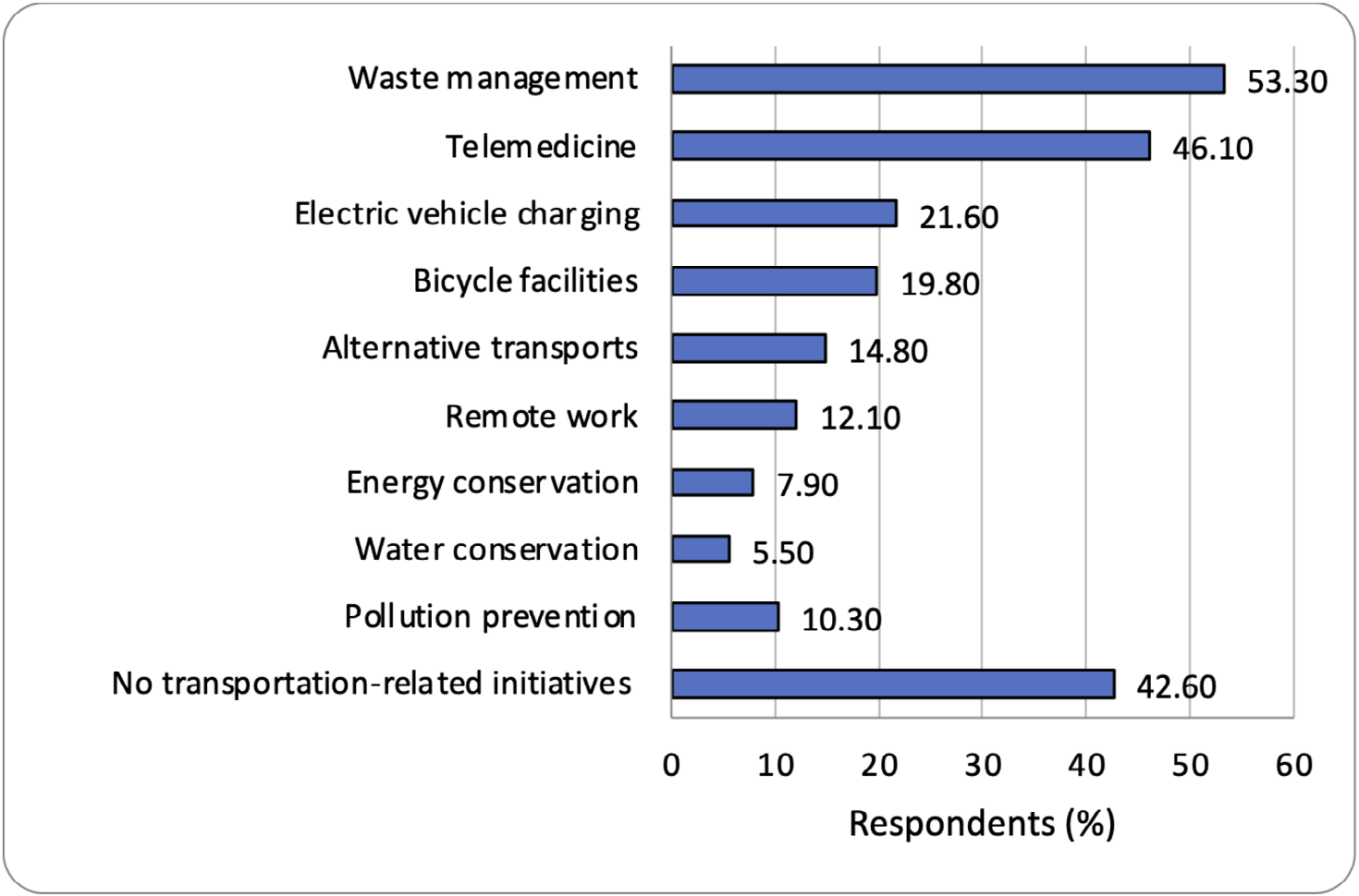
Reported institutional and workplace sustainability initiatives. Bars indicate the percentage of respondents reporting sustainability‐related initiatives in their workplace, including waste management, telemedicine, electric vehicle charging, bicycle facilities, alternative transport promotion, remote work, energy and water conservation, pollution prevention, and the absence of transportation‐related initiatives.

At the institutional level, 7.9% of hospitals engaged in energy conservation or renewable energy use, 5.5% practiced water conservation, and 53.3% implemented waste management. Pollution prevention measures were reported by 10.3%, and 46.1% utilized telemedicine, with 12.1% encouraging remote work. Transportation initiatives were less widespread, with 14.8% promoting alternative transportation modes, 19.8% providing bicycle facilities, and 21.6% installing electric vehicle charging stations. However, 42.6% reported no transportation‐related initiatives (Figure 2).

### 3.6 Modulators of Individual Opinions

We observed a clear hierarchy in risk perception. Respondents were significantly more concerned about long‐term societal impacts than immediate personal ones: Future Generations (3.80/4.0), Patient Health (3.18/4.0), Personal Impact (3.05/4.0). The overall Climate Concern Index, calculated as the arithmetic mean of the three variables, was 3.35/4.0. There was a high level of concern across the board, though “Specialists‐in‐training” (Residents) and core clinical neurologists showed the highest engagement. Adult Neurologists and Child Neuropsychiatrists showed remarkably consistent scores of 3.36/4.0, yet comparable with Pediatricians (3.11/4.0) and Epileptologists (3.19/40). The Climate Concern Index was quite homogeneous according to career stage (3.34–3.49/4.0) and geographically uniform across Italy, with only minor shifts between macro‐regions (3.32–3.36/4.0) (Table 2).

**TABLE 2:** Modulators of individual opinions.

		Mean CCI
Profession	Medical Residents	3.67
	Adult Neurologists	3.36
	Child Neuropsychiatrists	3.36
Career Stage	Early (< 7 years)	3.49
	Mid (7–15 years)	3.27
	Advanced (> 15 years)	3.34
Geography	Center	3.38
	South & Islands	3.36
	North	3.32

Abbreviation: CCI: Climate Concern Index (Scale 1–4).

## 4 Discussion

The study collected a moderately sized sample of 167 respondents, providing meaningful insights into the perspectives of epilepsy professionals in Italy on climate change and related professional practices. We observed near‐universal acknowledgment of global warming and high levels of concern about its consequences, particularly for future generations and for patients, mostly independent of profession, career stage, and workplace location. Respondents expressed uncertainty about whether society will successfully mitigate climate change, despite broadly agreeing that mitigation is achievable. A consistent pattern emerged across survey items: Strong perceived need for professional guidance and continuing education for patients and professionals on climate and health.

A comparable level of clinical awareness has previously been reported in studies focused both on non‐specialist healthcare professionals [10, 11], and among those involved in epilepsy care (patients, carers, clinicians) [9]. Of note, clinic administrators have expressed particular concern about the impact of acute weather events on frontline clinical activities, with barriers to preparedness including financing, knowledge, and resources [12]. High levels of concern about climate change's impact on future generations (100%), on oneself (83.9%), and on patients (84.3%) were even higher than reports from former studies, performed in non‐specialist health care workers [13]. The special vulnerability of people with seizures may drive a heightened concern in epilepsy professionals.

In spite of climate literacy, Italian epilepsy professionals reported uncertainty regarding humanity's capacity to address climate change effectively, pointing to a knowledge‐action gap [13, 14, 15, 16, 17]. Translating awareness into action requires not only a sense of individual self‐efficacy [18] but also adequate resources, training, and instruction, clear institutional leadership, support, and communication [19].

In this regard, official statements from scientific societies and targeted education strategies were considered particularly valuable, consistent with existing literature [20, 21]. Calls for enhanced climate‐health literacy have come from Italian medical students and residents [22] from professional societies such as the multinational Society of General Internal Medicine [23], from different specialists [24, 25]. There are no uniform education projects in this field, yet some preliminary findings merit consideration. Specific courses on Environmental Health and Medicine (EHM) focused on neurology have been carried out in Sweden, France, and Turkey, though their formal outcomes have yet to be evaluated [26]. A notable educational support can also come from meetings dedicated to climate and neuroscience, such as the Hot Brain conference, whose third edition was held in London in 2025 [27].

Live online competency‐based courses for professionals and patients have also been successful in fostering climate literacy [28, 29, 30]. Nevertheless, communication between patients and professionals on this topic remains underdeveloped and should be strengthened, with special attention to the lived expertise of people with epilepsy and their caregivers [9]. In a recent survey performed in Australia and New Zealand, only 32% of neurologists felt comfortable initiating conversations about climate change with patients, in spite of an overwhelming awareness of imminent threats [25].

Substantial gaps remain in the day‐to‐day environmental sustainability of clinical and research activities. Operational changes such as energy efficiency and waste management can meaningfully reduce healthcare's carbon footprint, while virtual conferencing has been shown to cut carbon emissions and energy use by over 90% [13, 31, 32, 33, 34, 35].

At the individual level, openness to innovative and dietary solutions exists, though infrastructural constraints, affordability concerns, and nutritional considerations—particularly in low‐ and middle‐income countries—limit their uptake. Technological innovation is broadly valued by the general population, people with epilepsy, carers, and professionals alike as a tool to address climate change [9, 36].

Engagement in green research practices was relatively low among respondents, likely reflecting insufficient institutional support, lack of dedicated training, and competing academic priorities. Practical steps—such as reducing freezer energy use, minimizing single‐use plastics, and optimizing laboratory workflows—are available but often hindered by safety requirements, equipment costs, and funding systems that do not currently reward sustainability [37].

In hospital settings, there are still no standardized sustainability metrics, which makes benchmarking difficult. Structured scoring frameworks covering energy, waste, water, emissions, and transportation could help track institutional progress. Nonetheless, telemedicine uptake—accelerated by the COVID‐19 pandemic—represents a meaningful step forward, reducing patient travel and associated emissions. Hospitals that have embedded sustainability into their operations report both greenhouse gas reductions and cost savings, strengthening the case for broader adoption [38, 39, 40].

Further applicable strategies to be evaluated could consist of expanding telemedicine for routine follow‐up, formalizing remote EEG reporting, and promoting home‐based or ambulatory EEG recording to reduce resource‐intensive inpatient stays [41, 42].

Several limitations of our study should be acknowledged. First, the use of a voluntary survey introduced selection bias, as participants were likely those with greater motivation or interest in the topic, potentially limiting the generalizability of findings. Second, reliance on self‐reported data may have introduced recall or reporting biases, as respondents' perceptions could differ from objective measures. Third, the sample exhibited uneven representation across professional roles and geographic regions, which may not fully reflect the broader European neurological community. Finally, the questionnaire employed was not formally validated, raising concerns about its reliability and ability to accurately capture the intended constructs. Development and validation of a dedicated, domain‐structured questionnaire, incorporating standard procedures such as expert panel review, cognitive interviewing, pilot testing, and reliability analysis, would represent an important next step for the field.

Further domains remain to be explored. Relevant seizure triggers could be further explored qualitatively through open‐ended questions, to capture individual stressors as well as complex multidimensional interactions that structured scales may fail to detect. For instance, the role of gut dysbiosis within a proposed “climate–gut–brain axis” [7], could be explored by specifically asking about changes in gastrointestinal patterns, including bowel habits, bloating, or dietary shifts during periods of climatic stress. Similarly, qualitative methods could help uncover the contribution of intercurrent psychosocial stressors, such as conflict, displacement, or climate‐related grief, which are unlikely to emerge spontaneously in structured interviews, yet may meaningfully compound biological vulnerability. It would also be noteworthy to enquire into the perceptions of the medication’s effectiveness or physical properties under extreme heat, as rising temperatures may compromise the pharmaceutical stability of antiseizure medications and alter their therapeutic impact [43, 44].

## 5 Conclusions

The survey of Italian epilepsy professionals suggests a meaningful awareness of climate change as a relevant health concern within the field. All respondents acknowledged global warming, and most perceived potential health impacts on themselves, their patients, and future generations, indicating a broadly shared recognition of the issue, though the extent to which this translates into clinical preparedness or practice change remains to be formally assessed.

Support for professional resources—including official statements by scientific societies and continuing education on climate and health—was notable among participants. Many also reported personal pro‐environmental behaviors such as waste reduction, energy conservation, and use of sustainable transportation, reflecting individual‐level engagement.

However, changes in research practices were reported to be limited, consistent with well‐documented systemic barriers to embedding sustainability within academic and clinical environments, and this finding should be interpreted cautiously, given the self‐reported nature of the data. Some institutional initiatives (e.g., waste management and telemedicine programs) were identified, suggesting early‐stage progress in select areas.

These findings point to a potential role for scientific societies and collaborative climate‐health research groups in supporting the field going forward. Possible contributions may include the development of guidelines, consensus statements, and multicenter studies with the active engagement of patients. Prospective studies designed to measure how epilepsy professionals’ climate literacy can translate into effective adaptation and mitigation strategies would be needed.

## Author Contributions

**Emanuele Bartolini:** conceptualization, methodology, investigation, formal analysis, writing – original draft, writing – review and editing, supervision. **Silvia Pradella:** investigation, resources. **Alessandra Rossi:** investigation, resources. **Giulia Battaglia:** investigation, data curation, writing – review and editing. **Pia Bernardo:** investigation, resources. **Annamaria Vezzani:** conceptualization, methodology, writing – review and editing, supervision. **Leila Zummo:** investigation, resources. **Simona Balestrini:** investigation, data curation, writing – review and editing. **Manuela Lo Bianco:** investigation, resources. **Giovanni Falcicchio:** investigation, resources. **Francesco Fortunato:** investigation, resources. **Teresa Ravizza:** investigation, resources. **Emilio Russo:** conceptualization, methodology, writing – review and editing, supervision.

## Funding

This work has been partially supported by grant‐RC, Italian Ministry of Health.

## Ethics Statement

The authors have nothing to report.

## Consent

The authors have nothing to report.

## Conflicts of Interest

The authors declare no conflicts of interest.

## Data Availability

The data that support the findings of this study are available from the corresponding author upon reasonable request.
